# Sequence and phylogeny of the complete mitochondrial genome of the Himalayan jungle crow (*Corvidae*: *Corvus macrorhynchos intermedius*) from Pakistan

**DOI:** 10.1080/23802359.2019.1704637

**Published:** 2019-12-18

**Authors:** Farheena Iqbal, Qasim Ayub, Beng Kah Song, Robyn Wilson, Muhammad Fahim, Sadequr Rahman

**Affiliations:** a School of Science, Monash University Malaysia, Bandar Sunway, Malaysia;; b Centre for Applied Molecular Biology, University of the Punjab, Lahore, Pakistan;; c Monash Genomic Facility, Monash University Malaysia, Bandar Sunway, Malaysia;; d Centre for Omic Sciences, Islamia College University, Peshawar, Pakistan;; e Tropical Medicine and Biology Platform, Monash University Malaysia, Bandar Sunway, Malaysia

**Keywords:** Mitochondrial genome, Himalayan crow, *Corvus macrorhynchos intermedius*

## Abstract

*Corvus macrorhynchos* formerly referred to as the jungle crow or the large-billed crow is a polytypic species with unresolved taxonomy, comprising various subspecies widespread across South, Southeast, and East Asia. In this study, we report the complete mitogenome of one of these subspecies, *Corvus macrorhynchos intermedius* (Himalaya crow), from Pakistan. The mitochondrial genome is circular, 16,927 bp and contains typical animal mitochondrial genes (13 protein-coding genes, 2 ribosomal RNA, and 22 transfer RNA) and one non-coding region (D-loop) with a nucleotide content of A (30.6%), T (24.8%), G (14.8%), and C (29.8%). Phylogenetic analysis using the whole mitochondrial genome showed that *C. m. intermedius* and only reported subspecies *Corvus macrorhynchos culminatus* (Indian Jungle crow) are genetically distinct and it supports the recognition of the latter as a separate biospecies.

*Corvus macrorhynchos sensu lato* (the jungle crow) is a species complex comprising 11–13 subspecies ranging in size, vocalization, and plumage (Dickinson et al. 2004; Clements et al. 2018; Del Hoyo et al. 2019). In the Indian subcontinent, three subspecies, *C. m. intermedius* (Adams, 1859), *C. m. culminatus* (Sykes, 1832), and *C. m. levaillantii* (Lesson, 1831) are recognized on the basis of their size, vocalization, and range. The comparatively larger sized *intermedius* (Himalayan crow) inhabits northwest Pakistan and Nepal along the Himalayas. The shorter sized *culminatus* (Indian jungle crow) resides in peninsular India and Sri Lanka, whereas *levaillantii* (Eastern jungle crow) is dispersed across Northeastern India, Bangladesh, and Southeast Asia (Burn and Madge 1999). Various ornithologists have suggested dividing these three regional subspecies into two or three species based on their interspecies differentiation. Martens et al. (2000) treated *culminatus* and *levaillantii* as distinct species splitting them from the *Corvus macrorhynchos* complex, while Rasmussen and Anderton (2005) proposed a further split into three correlating with their acoustic differences and morphology .

Recently, the IOC World Bird List granted a separate species status of monotypic *C. culminatus and C. levaillantii* while retaining all other subspecies including *C. m*. *intermedius* within *C. macrorhynchos* polytype (Gill and Donsker 2019). Thus, after this taxonomic upgradation, the previously reported mitogenome sequence from Sri Lanka (KR057957 and KR072661) may represent *C. culminatus* species (Krzeminska et al. 2016). This study would help in resolving the classification question and phylogeny within *C. macrorhynchos s.l.*

In this study, DNA was extracted from the feathers collected from a culled bird in Astor, Gilgit-Baltistan, Pakistan (35.33°N and 74.78°E) and stored in Monash University, Selangor, Malaysia (Accession No. Cmac-AS01). The whole mitochondrial genome was amplified as two overlapping PCR products that were sequenced using 250 bp paired-end Illumina MiSeq platform. The sequenced reads were assembled through MITObim (Hahn et al. 2013) and annotated by online MITOS software (Bernt et al. 2013).

The complete mitogenome of *C. m. intermedius* is 16,927 bp (GenBank Accession No. MN069302) comprising 37 genes (13 protein-coding genes, 2 ribosomal RNA genes, and 22 transfer RNA genes) and a putative long noncoding region called the control (D-loop) region, agreeing with the other *Corvus* gene arrangements.

The phylogenetic analysis (Figure 1) of *C. m. intermedius was* conducted by applying all available complete mitochondrial genomes from genus *Corvus* along with eight species of the same family, *Corvidae*. *C. m. intermedius* showed 97.5% identity with two linages of *C. m. culminatus* from Sri Lanka that is mutually 99.7% identical. The pairwise genetic distance among the two subspecies is 0.026 which is higher than distance (0.003) observed between the two *C. m. culminatus* mtDNA sequences and *C. splendens* subspecies and corresponds closely with the interspecies distance between *Corvus corax* and *Corvus cryptoleucus* (0.032). Irrespective of the small representation of all described subspecies, this genomic study supports the proposed species-level distinction of *C. culminatus* and also provides additional data for deducing intraspecific taxonomy, speciation, and phylogeography of *C. macrorhynchos s.l.* complex.

**Figure 1.**
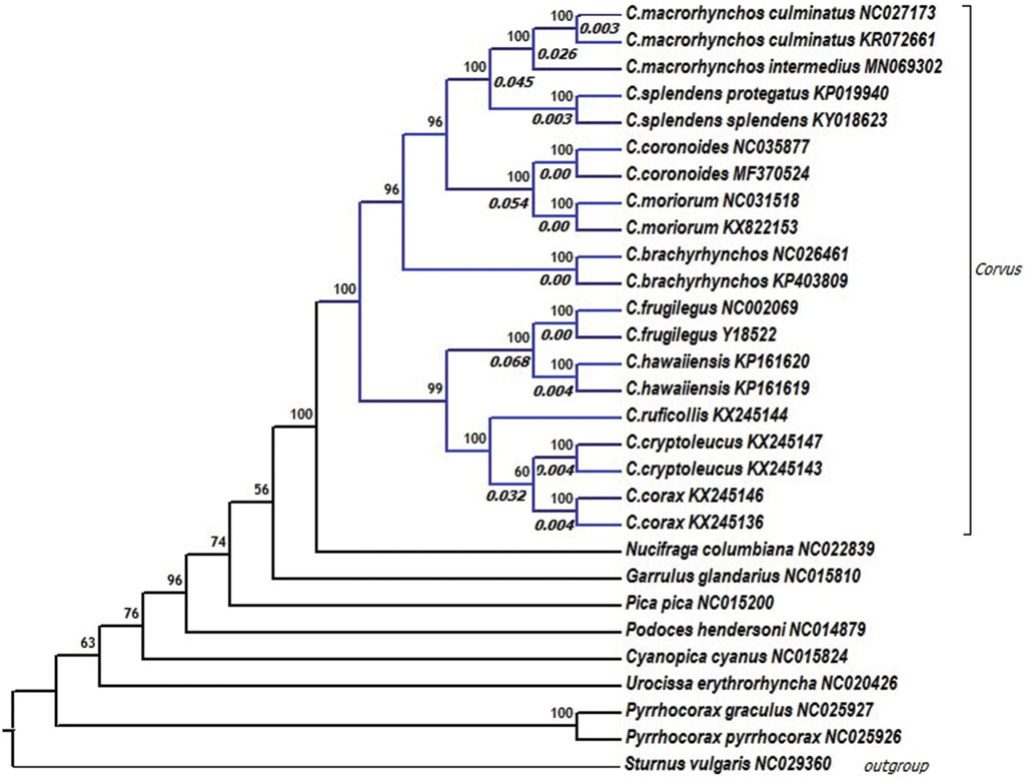
Phylogenetic relationship of genus *Corvus* within family *Corvidae* using *Sturnus vulgaris* (family: *Sturnidae*) as outgroup. The phylogenetic tree was derived from complete mitochondrial genome sequences and constructed using a maximum likelihood method with 1000 bootstrap replicates in the program RAxML (Stamatakis 2014). Numbers beside the branch point indicate bootstrap support values and those in italics indicate the pairwise genetic distance among the mitochondrial lineages estimated by MEGA7 (Kumar et al. 2016) using the Tamura–Nei model. Alphanumeric codes after the species name indicate the GenBank accession numbers.
